# Abnormal Food Timing Promotes Alcohol-Associated Dysbiosis and Colon Carcinogenesis Pathways

**DOI:** 10.3389/fonc.2020.01029

**Published:** 2020-07-17

**Authors:** Faraz Bishehsari, Shirin Moossavi, Phillip A. Engen, Xiaohan Liu, Yue Zhang

**Affiliations:** ^1^Department of Internal Medicine, Division of Gastroenterology, Rush University Medical Center, Chicago, IL, United States; ^2^Department of Medical Microbiology and Infectious Diseases, University of Manitoba, Winnipeg, MB, Canada; ^3^Children's Hospital Research Institute of Manitoba, Winnipeg, MB, Canada; ^4^Digestive Oncology Research Center, Digestive Disease Research Institute, Tehran University of Medical Sciences, Tehran, Iran; ^5^Department of Mathematics, Statistics, and Computer Science, University of Illinois at Chicago, Chicago, IL, United States

**Keywords:** alcohol, colon cancer, circadian rhythm, food timing, gene expression, microbiota

## Abstract

**Background:** Alcohol consumption is an established risk factor for colorectal cancer (CRC). Identifying cofactor(s) that modulate the effect of alcohol on colon inflammation and carcinogenesis could help risk stratification for CRC. Disruption of circadian rhythm by light/dark shift promotes alcohol-induced colonic inflammation and cancer. More recently, we found that abnormal food timing causes circadian rhythm disruption and promotes alcohol associated colon carcinogenesis. In this study, we examined the interaction of wrong-time feeding (WTF) and alcohol on CRC-related pathways, in relation to changes in microbial community structure.

**Methods:** Polyposis mice (TS4Cre ×*cAPC*^Δ468^) underwent four conditions: alcohol or water and feeding during the light (wrong-time fed/WTF) or during the dark (right-time fed). Colonic cecum mucosal gene expression was analyzed by RNA-seq. Microbiota 16S ribosomal RNA sequencing analysis was used to examine colonic feces. Modeling was used to estimate the extent of the gene expression changes that could be related to the changes in the colonic microbial composition.

**Results:** The circadian rhythm pathway was the most altered pathway by the WTF treatment, indicating that WTF is disruptive to the colonic circadian rhythm. Pathway analysis revealed interaction of WTF with alcohol in dysregulating pathways related to colon carcinogenesis. Similarly, the interaction of alcohol and WTF was detected at multiple parameters of the colonic microbiota including α and β diversity, as well as the community structure. Our modeling revealed that almost a third of total gene alterations induced by our treatments could be related to alterations in the abundance of the microbial taxa.

**Conclusion:** These data support the promoting effect of abnormal food timing alcohol-associated CRC-related pathways in the colon and suggest colon dysbiosis as a targetable mechanism.

## Introduction

Colorectal cancer (CRC) is the third most common cancer in the world (1). Among the lifestyle habits, alcohol consumption is an established factor that increases the risk of colonic premalignant as well as malignant lesions (CRC) (2, 3). Nonetheless, only a small portion of alcohol consumers develop CRC, suggesting that additional cofactor(s) may promote CRC-related mechanisms in the setting of alcohol intake. Circadian rhythm disruption is another lifestyle habit, increasingly common in our modern societies (4). Under homeostasis, circadian clocks control rhythms of physiologic processes in our organs, which if disrupted can predispose to chronic pathologies (5). A remarkable portion of modern societies is exposed to some form of circadian rhythm disruption such as light/dark shifting, long travels, irregular work schedules, or social jet lag (6). Another less studied form of circadian rhythm disruption, particularly relevant to the intestine, is abnormal eating patterns (7). Earlier animal experiments indicated that time-restricted feeding at the wrong/rest time can shift the circadian oscillation of the colon clocks independently of the effect of light, leading to circadian dyssynchrony (8, 9). Shift in the time of meal not only disturbs the circadian rhythmicity, but also could promote metabolic and behavioral alterations in animal studies (10).

Interaction of circadian rhythm disruption with alcohol on intestinal pathologies is supported by prior studies from our group (11–14). For example, alcohol-induced intestinal barrier dysfunction, a feature that promotes mucosal and colonic inflammation and cancer (15), is augmented in the setting of light dark shifting (13). Specifically, in CRC, we also found that light/dark shifts promoted alcohol-induced polyposis and colon cancer, an effect that was associated with changes in microbiota (11). Accumulating evidence suggests a remarkable role for microbiota in colonic inflammation and carcinogenesis (16). Gut microbiota modulates gene expression and the cancer-related pathways in the colon (17). Each alcohol intake or light/dark shifting could individually change the gut microbiota composition, but the microbiota effect is enhanced if the two are combined (18, 19). It has been recently shown that abnormal food timing also resulted in dramatic changes in microbiota (20). We recently reported on the interaction of abnormal food timing (eating during the rest phase: wrong time eating), with alcohol consumption on colon polyposis and CRC (21).

In this study, we examined the interaction of wrong-time feeding (WTF) and alcohol on CRC-related pathways in a colon-specific polyposis mouse model (11). We hypothesized that the WTF would affect circadian rhythm pathways in the colon and elevate alcohol-associated dysregulated genes in promoting CRC susceptibility. To elucidate possible mechanisms, we assessed the interactions of alcohol with WTF on colonic microbiota. We found that WTF dysregulated circadian rhythm pathways in the colon, and promoted alcohol-induced colon cancer–related pathways that also coincided with an altered colonic microbiota community structure. Our results indicate that altered colonic gene expression may be in part related to the microbial changes imposed by WTF and alcohol.

## Materials and Methods

### Animals

**Figure 1:**
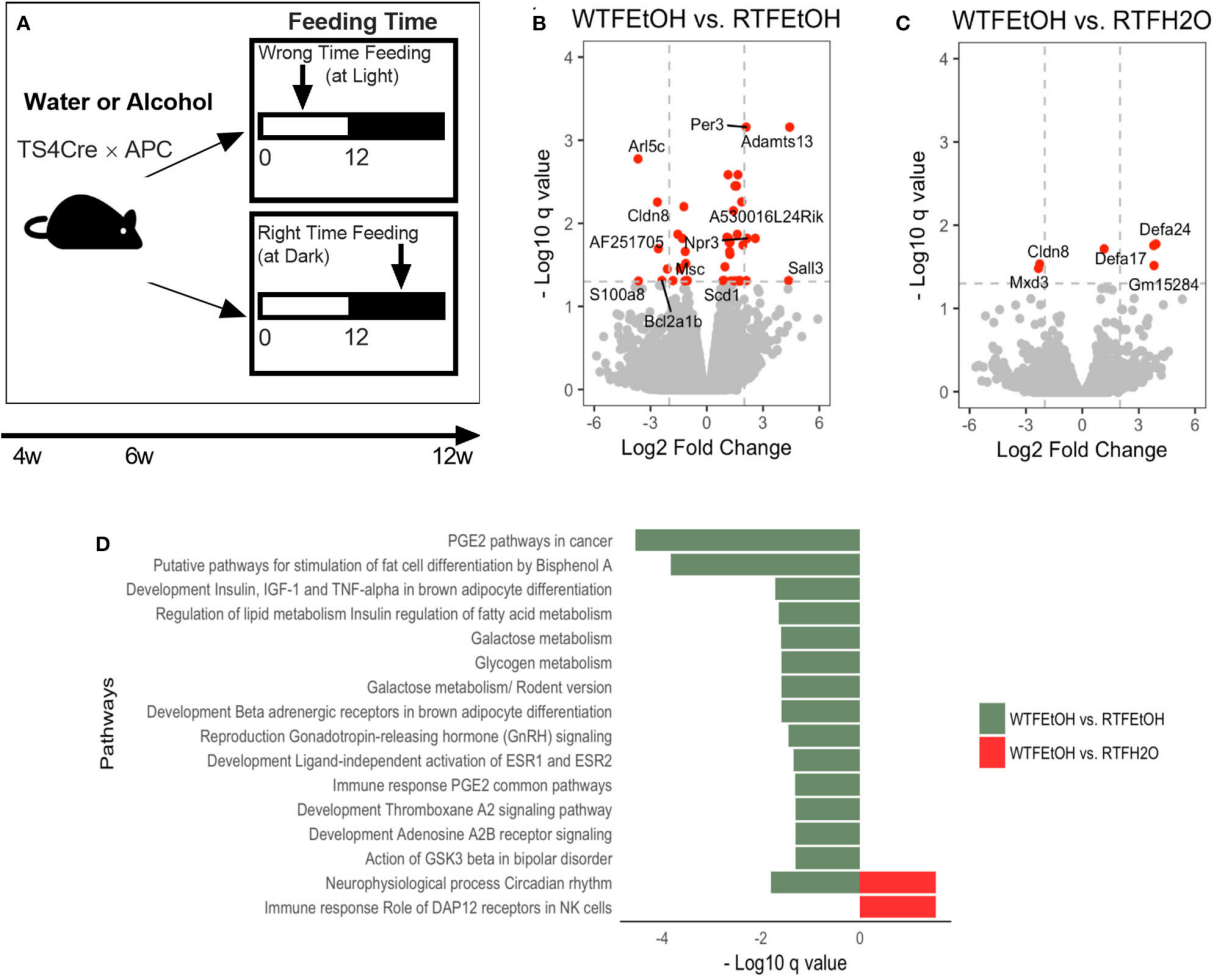
Impact of concomitant alcohol consumption and circadian rhythm disruption on host gene expression. **(A)** Mice underwent (right-time feeding/RTF during the dark vs. wrong-time feeding/WTF during the light) with or without alcohol. Alcohol dosing was gradually increased over 2 weeks to the goal of 15%. Alcohol was provided during the feeding time. **(B)** Gene expressions significantly (FDR-*p* < 0.05) altered in alcohol + WTF compared to alcohol + RTF and **(C)** water + RTF using two-way ANOVA; **(D)** Significant (FDR-*p* < 0.1) gene pathway enrichment analysis. The significant differentially expressed genes were put through pathway map analysis using MetaCore. The enriched pathways were ranked according to their FDR, representing the most significant DEGs in group pairwise comparisons. RTF, right-time feeding; WTF, wrong-time feeding.

To model polyposis, the TS4Cre mice were crossed with LoxP APC^Δ468^ mice at Rush University Medical Center (IACUC # 14-008) to generate the TS4Cre × cAPC^Δ468^ mice, as previously reported (11, 22). Mice were maintained on a regular 12-h light/12-h dark cycle; zeitgeber time (ZT) 0 and ZT12 are the times that light turns on and off, respectively. Mice (*n* = 5 per group) at 4 weeks of age were treated with *ad libitum* chow diet under two food timing conditions [right-time feeding (RTF) during the dark vs. WTF during the light] with or without alcohol. Alcohol was provided during the feeding time. Alcohol dosing was gradually increased over 2 weeks to the goal of 15% (23), as previously described (Figure 1A). To resemble human conditions, mice were given *ad libitum* access to food and water during the weekends. Mice were sacrificed after 3 months at the same ZT (0) with respect to the zeitgeber timing, and the colonic feces were immediately collected for microbial analysis. This protocol was associated with an overall comparable alcohol intake among the groups, while resulting in a worsening alcohol-induced polyposis by WTF (Figure S1), as previously described (21). In order to eliminate the immediate effect of time of food intake on the gene expression, animals had free access to food and water 24 h before the sacrifice. This ensured that the gene expression changes observed in this study did not reflect the immediate effect of food availability. We did not use wild-type mice, as our prior studies indicated a significantly different microbial composition in these mice from the polyposis model, limiting their utility for interrogating microbiota changes in comparison to polyposis model (22). All experiments were conducted at Rush University Medical Center with approval of the Institutional Animal Care and Use Committee.

### RNA Preparation and RNA-Seq Quantification

For the RNA-seq run, animals underwent the combination of control feeding (RTF) vs. WTF, with water or alcohol treatment. The cecum mucosa was immediately harvested and frozen for RNA extraction. Total RNA was extracted from using the Maxwell® 16 LEV simplyRNA Tissue Kit (Promega, Madison, WI, USA). RNA was quantified in a Qubit 3.0 fluorometer using the RNA BR assay kit (Thermo Fisher Scientific, Grand Island, NY, USA). RNA quality was assessed using an RNA ScreenTape kit on 2200 TapeStation System (Agilent, Palo Alto, CA, USA). RNA-seq libraries were prepared using the Lexogen QuantSeq 3′ mRNA sequencing library preparation kit for Illumina sequencing platform-compatible libraries. Upon verification of the library quality, libraries were run on an Illumina Nextseq500 instrument, using the high-output (1 × 75 cycle) kit, with read lengths of 80 bases at the University of Illinois at Chicago (UIC) Core Genomics facility, and sequencing was performed in the DNAS facility [Research Resources Center (RRC), UIC].

Burrows–Wheeler Aligner (BWA MEM) (24) was used to align the reads, and FeatureCounts (25, 26) was used to quantify the genes expression levels. To ensure high-quality data, quality-control checks were performed to exclude sequencing artifacts and to include sufficient reads in coding sequences to increase accuracy of expression quantifications.

### RNA-Seq Bioinformatics Analysis

The raw 3′ mRNA reads of each sample were aligned to the mm10 reference genome using BWA MEM (24), which efficiently maps reads with read-through into polyA tails and adapter sequences, as is common with 3′ RNA-seq. The expression levels of genes were quantified using FeatureCounts (25, 26). One sample was excluded from further analysis due to low quality. Next, edgeR was used to perform differential analysis given quantified raw counts of reads (27, 28). Using two-way analysis of variance (ANOVA), we determined the genes that were significantly affected by our treatments (alcohol, or WTF, and their interactions). An adjusted *p*-values (*q*-value) threshold of 0.05, corresponding to a false discovery rate (FDR) of 5%, was considered to define significance.

The significant differentially expressed genes (DEGs) were identified for omnibus and pairwise comparisons. Significant DEGs obtained through pairwise comparison between the groups treatments (e.g., WTF + water group vs. RTF + water group) were put through pathway map analysis using MetaCore (https://clarivate.com/cortellis/solutions/early-research-intelligence-solutions/). Pathways with FDR <0.1 were enriched; the enriched pathways were ranked according to their FDR, representing the most significant DEGs in group pairwise comparisons.

### DNA Extraction and Microbiota Analysis

Total DNA was extracted from colonic feces utilizing FastDNA bead-beating Spin Kit for Soil (MP Biomedicals, Solon, OH, USA). High-throughput amplicon sequencing was conducted using primers (515F/806R) targeting variable region 4 (V4) of the 16S ribosomal RNA (rRNA) genes (29), in a modified two-step targeted amplicon sequencing approach (30). Sequencing was performed using an Illumina MiSeq (Illumina, San Diego, CA, USA) at the Sequencing Core of the University of Illinois at Chicago, as previously described (11, 21, 22).

Data processing, quality control, and biological observation matrix analysis were performed, as previously reported (21, 31–34). Briefly, raw FASTQ files for each sample were processed using the software package PEAR (paired-end read merger) (v0.9.8) (31). Subsequently, data were imported into CLC Genomics Workbench (v10.0) (CLC Bio; Qiagen, Boston, MA, USA) and (1) primer sequences were removed, (2) sequences without both forward and reverse primers were discarded, and (3) sequences were trimmed using quality trimming (quality threshold, Q20) and length trimming (discarding everything <250 bp) algorithms. The trimmed files were then exported as FASTA files into the QIIME (v1.8) (32) for chimera removal using the USEARCH (v8.1) algorithm (33). The chimera-free FASTA files were then processed to cluster sequences into operational taxonomic units (OTUs) at a similarity threshold of 97% using the UCLUST algorism method. Representative sequences for each OTU were selected, and these sequences were annotated using the UCLUST and the Greengenes_13_8 reference (97_otus.fasta) and taxonomy database (97_otu_taxonomy.txt) (34). The demultiplexed sequencing data were deposited into the Sequence Read Archive of NCBI and can be accessed via accession number PRJNA523141.

Initial preprocessing of the OTU table was conducted using the Phyloseq package (35). Briefly, samples were rarefied to the minimum 43,000 sequencing reads per sample. The numbers of sequencing read per taxa were relativized to the total sum of 43,000 without excluding rare taxa. Overall, 55,295 unique OTUs were detected. Operational taxonomic units belonging to archaea, family of mitochondria, and class of chloroplast were excluded from the analysis. Operational taxonomic units with fewer than five reads across the entire dataset were removed, resulting in 1,564 remaining OTUs. This dataset was used for analysis unless otherwise specified. The contribution of the excluded rare OTUs to the total reads per sample was deemed negligible (Figure S2). Within-sample (α diversity: inverse Simpson index and richness) and between-sample (β diversity) are used to show differences in microbial community structure, as described below.

### Microbiota Comparisons

Data analysis was conducted in R (36). All statistical analyses were performed after excluding OTUs with fewer than five reads across the entire dataset unless specified otherwise. α diversity was assessed by the observed OTUs (richness) and inverse Simpson Index (diversity). Association of α diversity with alcohol consumption, LD cycle, and their interactions was assessed by linear regression. Subgroup analysis was performed using ANOVA with *post-hoc* pairwise comparison. Dissimilarity (β diversity) of clusters was assessed on Bray–Curtis dissimilarity distance by permutational ANOVA (PERMANOVA) using the vegan package (37). *Post-hoc* pairwise comparisons were conducted using pairwise Adonis package (38). To control for the compositional nature of the data, OTU counts were center log-ratio transformed following zero replacement (39, 40). After this transformation, taxa relative abundances were compared at phylum, genus, and species levels by linear regression. The *p*-values were corrected with Benjamini–Hochberg's FDR method (41).

### Microbiota–Gene Expression Correspondence Analysis

To test whether the effect of alcohol intake and abnormal food timing (WTF) on the colonic gene expression could be at least partly explained by changes in microbiota, we correlated the two obtained datasets: microbiota and the gene expression datasets. As the experiments to collect RNA and the microbiota had to be performed on two separate runs of animals, and in order to boost our analysis, we first performed a dimension reduction on the two datasets in preparation for correlation analysis, as reported in the following sentences.

Given the remarkable difference of microbiome taxa counts across groups, as well as within groups, we focused our analysis on the abundant taxa, by including taxa whose average counts were higher than 10 for all four mice groups. In this way, we could reduce the microbiome dataset dimension from 211 to 34 taxa. Further standardization was done by converting count data to percentage data because of its good property of continuity. To include commonly expressed genes in our gene expression dataset, we first removed genes with more than one record of “0” in their relative abundance. On the remaining 13,782 genes, we performed normal standardization. As a result, we had 34 microbiome taxa and 13,782 genes to be used in the correspondence analysis.

Next, we aim to examine the effects of experimental factors (alcohol and WTF) and microbiota on the colonic gene expression. Because the RNA and microbiota data were collected on two cohorts of mice with the same linage, we first randomly paired up one observation from microbiota data with one observation from gene expression data in the same treatment group for all the four treatment groups. This yielded in multiple records of arbitrarily assigned 1-to-1 data pairs in RTF/water, RTF/alcohol, WTF/water, and RTF/alcohol groups.

Then, we repeated the gene/microbiome pairing procedure for 500 times on each of the 34 abundant genes with the same random seed and the times when one microbiome having a significant effect on a gene expression could be counted. Under the Bonferroni correction with α = 0.05, We assumed that any gene/microbiome pair having significant effects occurring more than 53 times out of 500 could be considered as candidates whose relationships were likely to be resistant to the lack of 1-to-1 relationship.

Eventually, we built a two-step linear regression to identify the association between gene and taxa alterations due to the interaction of experimental factors on genes with significant effects. To this end, we used *F*-test to test the null hypothesis that the inclusion of the 34 microbiome taxa (predicting factors) to our treatments (alcohol vs. water and WTF vs. RTF) would not affect the colon gene expression (the outcome). A rejection of the null hypothesis would suggest that changes in the colon microbiota could explain changes in the colon gene expression in our model. We calculated the number of rejection counts yielded out of the total 500 random one-to-one correspondence events. Because the larger the rejection count is, the stronger the null hypothesis is rejected, we included only genes with rejection count of more than 40 to our model. In total, 369 genes out of 13,782 had rejection counts of more than 40 under our model. Mean expression levels of these genes within different experimental groups are presented in a heatmap. Relative fold change within each row is visualized by dividing the value by the mean of values in the row.

The 369 genes were used to build our two-step regression models. In the first model *M*_*l*_ = β_0_+ β_1_*X*_1_+β_2_*X*_2_+ β_3_*X*_1_*X*_2_+ ε_*l*_, where *l* is the number of taxa, *X*_1_ accounts for alcohol/water consumption, and *X*_2_ accounts for right of WTF, we identified taxa with significant *p*-values for the interaction term β_3_*X*_1_*X*_2_ (*p* < 0.05). The model allowed us to select taxa where the taxa were significantly affected by our treatment (alcohol and/or food timing). Then, the selected taxa and taxa–treatment interactions were brought into the second model to identify the genes that were significantly affected by at least one treatment and one selected microbial taxa. In this second model, *Y*_*kj*_ = β_0_+ β_1_*X*_1_+β_2_*X*_2_+ β_3_*X*_1_*X*_2_+ β_4_*M*_*t*_+ β_5_*M*_*t*_*X*_1_*X*_2_+ ε_*kj*_, *j* is the mice, *k* index of genes (369 in total)*, t* index of taxa identified by model *1*, and *M*_*t*_is the *t*th taxa, so model 2 is constructed taxa by taxa. *X*_1_ accounts for alcohol/water consumption, and *X*_2_ accounts for right of WTF. We then identified genes with significant association with both the interaction term (β_3_*X*_1_*X*_2_) and taxa (β_4_*M*_*t*_).

## Results

### Alcohol Consumption and/or WTF Altered Colonic Gene Expression

Alcohol or WTF each separately affected the cecum mucosa gene expression levels (Figures S3A,B). Alcohol alone resulted in two-fold down-regulation of *Clps* (colipase), *Cpa2* (RNA polymerase II C-terminal domain phosphatase-like 2 in *Arabidopsis*), and *Frs3* (fibroblast growth factor receptor substrate 3) and up-regulation of *4930552P12Rik* (lincRNA) (Figure S3A). Wrong-time feeding alone resulted in significant up-regulation of *Ciart* (circadian-associated transcriptional repressor) and *Mterf1a* (transcription termination factor 1a, mitochondrial) (Figure S3B). Wrong-time feeding was sufficient to alter *Ciart* gene expression in comparison to alcohol alone (Figure S3C). Relative to each alone, concomitant alcohol intake and WTF had a stronger effect on the number of affected genes by treatment; the number of dysregulated genes in the alcohol + WTF group was the highest in comparison to other groups (Figures 1B,C). Several genes were up-regulated more than two-fold as a result of alcohol + WTF, including *Per3* (period circadian protein homolog 3) and *Adamts13* (a disintegrin and metalloproteinase with thrombospondin motifs 13), whereas *Arl5c* (ADP-ribosylation factor-like protein 5C) and *Cldn8* (claudin-8) were down-regulated.

Pathways analysis was performed to determine the most dysregulated pathways based on DEGs from our treatment(s); interestingly, comparing WTF vs. RTF mice that were treated by water, the circadian rhythm pathway was the most altered pathway by the WTF (Figure S4A). This confirms that WTF altered the colonic circadian rhythm as hypothesized. Pathway analysis also revealed interaction of alcohol and wrong time eating; comparing mice that consumed alcohol under wrong vs. RTF protocol revealed the greatest number of altered pathways in the colon. The top differentially expressed pathways belonged to the immune and cancer as well as metabolic pathways (Figure 1D, Figures S4B,C). This observation was interesting and is in line with the growing evidence that altered lipid metabolism is frequently seen in CRC and may be the gate for the environmental effects on colon carcinogenesis (42). In summary, we found that WTF altered circadian rhythm pathways in the colon and interacted with alcohol to dysregulate pathways related to inflammation and carcinogenesis in colon. This was in line with our recent observation that alcohol and WTF promote colon inflammation and carcinogenesis (21).

Intestinal gene expression has been shown to be affected by the microbial composition (17). Positive interactions of circadian disruption and alcohol have been previously reported by our group (11–13, 43, 44). Therefore, we next studied the colonic fecal microbiota that was in direct contact to the colonic cecum mucosa used for the gene expression analyses.

### Interaction of WTF With Alcohol Consumption on Colonic Microbiota Diversity and Composition

**Figure 2:**
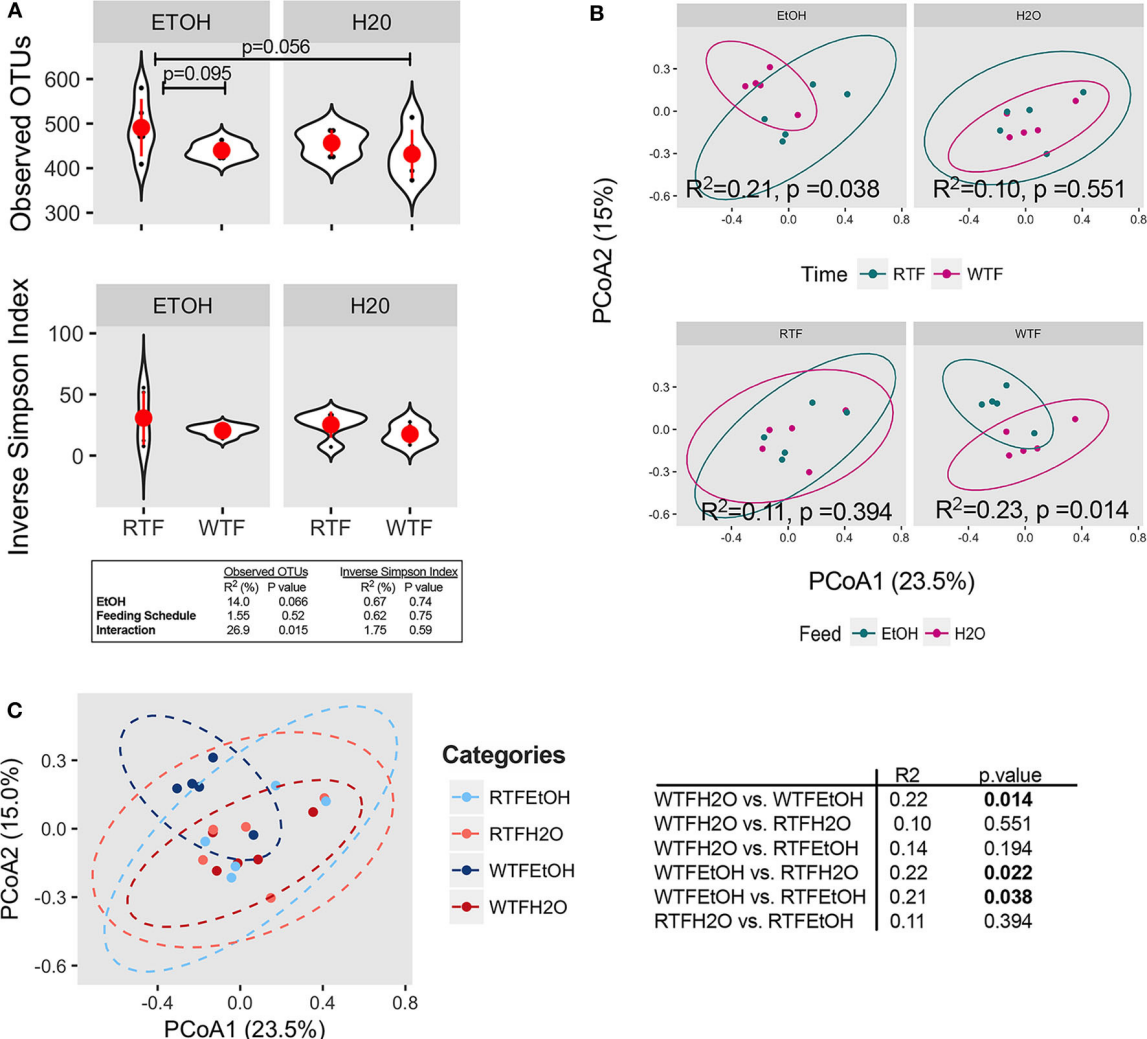
Concomitant alcohol consumption and circadian rhythm disruption alter gut microbiota diversity and overall composition. **(A)** α diversity assessed by the number of observed OTUs and inverse Simpson index across treatment categories; **(B)** β diversity assessed on Bray–Curtis dissimilarity matrix across treatment categories. α diversity was assessed using ANOVA, whereas β diversity was tested using PERMANOVA. Subsequently, pairwise comparisons were performed. *R*^2^ and *p*-value depicted within graphs. **(C)** Principal coordinates analysis (PCoA1 23.5% vs. PCoA2 15.0%) represents the overall gut microbial communities between treatment groups. Significantly different microbial compositions between mouse treatment groups were as follows: alcohol + WTF vs. WTF (*R* = 0.22, *p* = 0.014); alcohol + WTF vs. RTF (*R* = 0.22, *p* = 0.022); and alcohol + WTF vs. alcohol + RTF (*R* = 0.21, *p* = 0.038).

The average microbial richness was overall lower in mice fed during the wrong time when compared to the mice fed during the right time (Figure 2A), with WTF in the control (water-treated) group resulting in lower observed OTU richness compared to RTF in the alcohol group (*p* = 0.056). While we observed no significant effect of alcohol consumption or food timing alone on the microbiota α diversity (Figure S5), there was a significant interaction of the two factors in lowering the microbial observed OTU richness (*R*^2^ = 0.27, *p* = 0.015, Figure 2A). Simpson diversity index was not affected by either of the treatments. Next, we assessed the effects of alcohol consumption and WTF on the overall microbiota composition. There was no significant effect of alcohol or WTF alone on the overall microbiota composition (Figures 2B,C). However, consistent with their interaction effects on microbiota, we observed a significant difference in the overall composition according to WTF in the alcohol group (*R*^2^ = 0.21, *p* = 0.038) and according to alcohol consumption in the WTF group (*R*^2^ = 0.23, *p* = 0.014, Figure 2B).

### Impact of Alcohol Consumption and WTF on the Colonic Microbiota Structure

**Figure 3:**
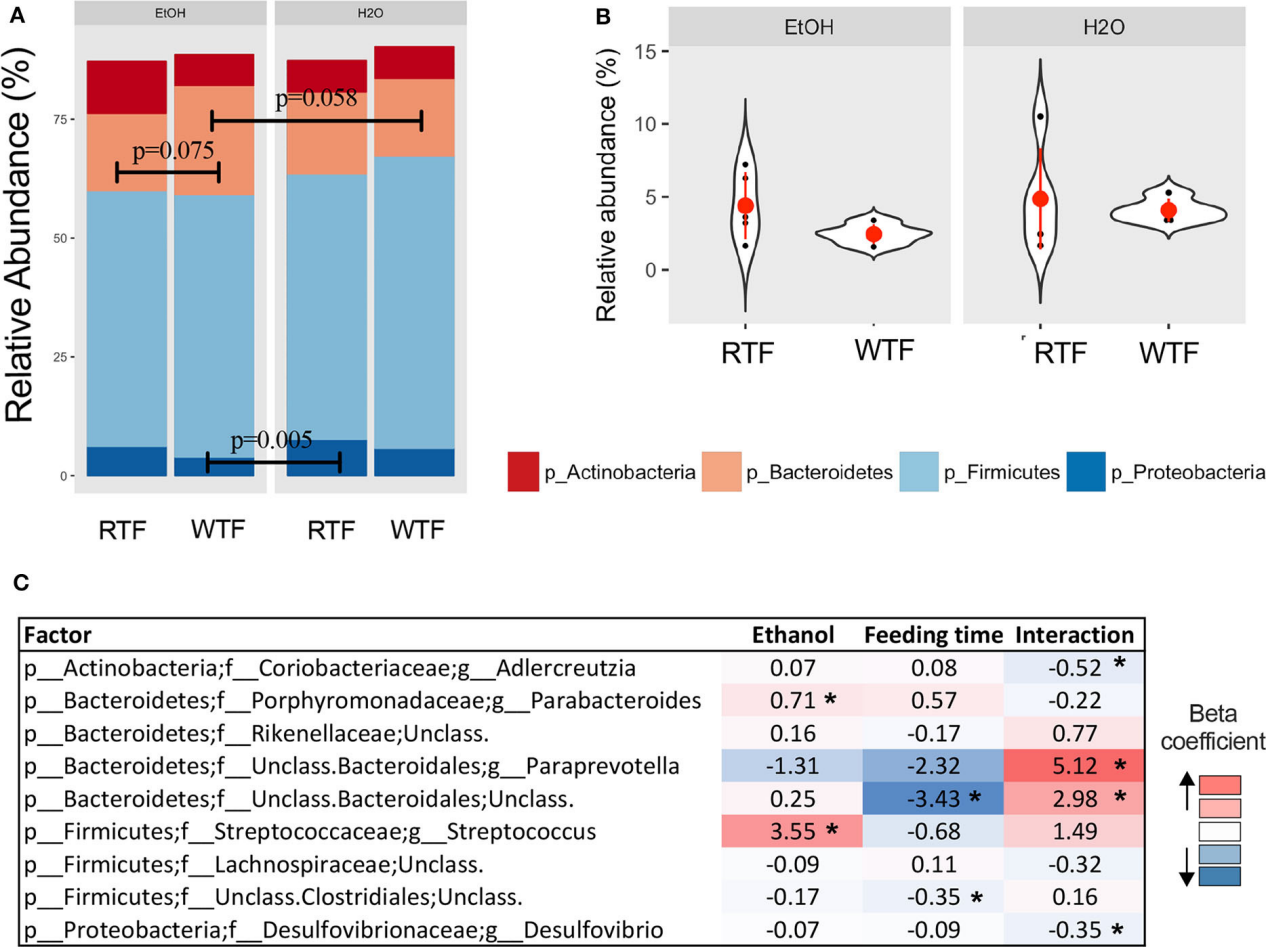
Impact of concomitant alcohol consumption and circadian rhythm disruption on gut microbiota structure. **(A)** Relative abundances visualized at phylum level; **(B)** Firmicutes-to-Bacteroidetes ratio across treatment categories (no significant differences); **(C)** Significant association of genus taxa with the interaction of alcohol consumption and circadian disruption assessed by linear regression. (*p < 0.05). β with * means that effect size is statistically significant with a *p*-value = 0.05.

Firmicutes, Bacteroidetes, Proteobacteria, and Actinobacteria were the predominant phyla regardless of the treatment (Figure 3A). Overall, there were trend differences in the relative abundance of Bacteroidetes between alcohol + WTF vs. alcohol + RTF (*p* = 0.075), as well as alcohol + WTF vs. water + WTF (*p* = 0.058). The relative abundance of Proteobacteria was significantly lower in alcohol + WTF compared to water + RTF (*p* = 0.005, Figure 3A). The Firmicutes-to-Bacteroidetes ratio was not significantly affected by either of the treatments (Figure 3B).

Alcohol consumption was associated with increased relative abundance of phylum Bacteroidetes (*p* = 0.007) and lower relative abundances phyla Firmicutes (*p* = 0.009) and Verrucomicrobia (*p* = 0.026). At the lower taxonomic levels, alcohol consumption was associated with significantly higher relative abundances of family Turicibacteraceae (*p*_adjusted_ < 0.001) and genus *Turicibacter* (*p*_adjusted_ < 0.001; Table S1). Wrong-time feeding was associated with increased relative abundances of phyla Bacteroidetes (*p* = 0.005) and TM7 (*p* = 0.02) and significantly lower relative abundance of phylum Firmicutes (*p*_adjusted_ < 0.03). At the genus taxonomic level, *Bacteroides* had an increased relative abundance in WTF (*p* = 0.005; Table S1).

In the multivariable linear regression, several taxa were associated with either alcohol consumption or WTF, whereas genera *Adlercreutzia* [$\hat{\beta }$ = −0.52, 95% confidence interval (CI) (−0.86 to −0.19)], *Paraprevotella* [$\hat{\beta }$ = 5.12, 95% CI (1.58–8.65)], unclassified Bacteroidales [$\hat{\beta }$ = 2.98, 95% CI (0.57–5.40)], and *Desulfovibrio* [$\hat{\beta }$ = −0.35, 95% CI (−0.66 to −0.03)] were associated with the interaction term (Figure 3C).

### Effect of Alcohol Consumption and WTF on the Colon Gene Expression Is Partly Explained by the Changes in the Colon Feces Microbiota

Intestinal microbial composition has been shown to affect intestinal gene expression (17). Given the positive interaction of alcohol and WTF on both colonic gene expression and microbiota, here we examined whether changes in the colonic microbial profiles might mediate our treatment effects on the colonic gene expressions. As explained in the *Materials and Methods*, we first created a one-to-one correspondence of the two datasets based on the hypothesis that interindividual variability accounts for the variation seen in the gene expression. Among the 13,782 genes (see *Materials and Methods*), 3,999 (~29%) had at least one paired microbiome significance (Table S2).

**Figure 4:**
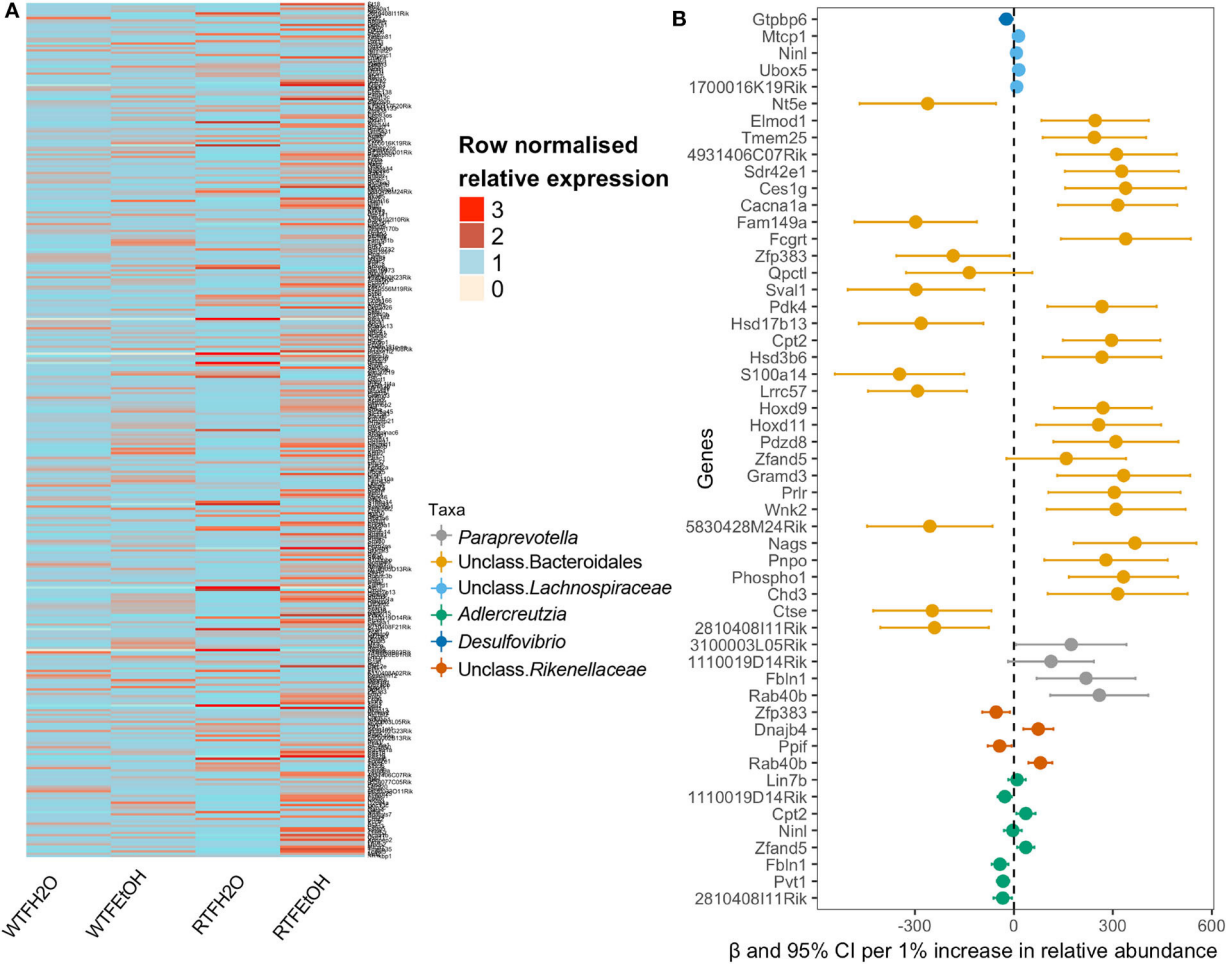
Identifications of gene expressions due to the additive effect of alcohol and circadian disruption, which are significantly associated with gut microbiota composition. **(A)** Heatmap of the selected genes in the on-to-one correspondence analysis; data are normalized within each row by dividing the value by the mean of values in the row. **(B)** Forest plot of the significant association of genus taxa with gene expression.

In order to examine if microbial differences could explain the alterations in the colon gene expressions, because of the interaction of alcohol consumption and WTF, we used a two-step regression model. As our model outcome, we selected 369 genes whose alterations were most likely not affected by random one-to-one correspondence (see *Materials and Methods*). The differential expression pattern of genes could separate the treatment groups as presented in Figure 4A. Our first-step model aimed to identify taxa (among 34 microbial taxa counts) that are significantly affected by the treatments (alcohol, food timing, and their interaction). Consistent with the significant interaction of alcohol consumption and abnormal food timing on microbial composition, six taxa were found to be strongly altered by the interaction of alcohol and WTF, whereas only three taxa were altered because of each of the factors alone (Table S3). These taxa could explain 32% of total gene alterations (119/369 genes) induced by our treatment. To infer specific microbe–gene axes that were likely due to the interaction of alcohol and abnormal food timing, we found 45 genes (38% of the 119 genes that were related to the microbial alterations) to be likely related to the changes in the six microbial taxa that were strongly altered by interaction of alcohol and WTF (Figure 4B).

## Discussion

Chronic alcohol consumption and circadian rhythm disruption are two of the known factors influencing gastrointestinal tract health and diseases (5, 45, 46), and each separately is shown to increase the risk of CRC (47–49). In a colon cancer model, we previously uncovered the interaction of circadian disruption by light/dark shift on the dysbiotic impact of chronic alcohol consumption and colon cancer formation (11). We recently showed that abnormal food timing, as defined by eating during rest (wrong time), induced central–peripheral circadian dyssynchrony, and promoted colon carcinogenesis in colon-specific polyposis mice that consumed alcohol (21). Here, using the same mouse model, we examined the interaction of WTF and alcohol on CRC-related pathways. We found that WTF dysregulated circadian rhythm pathways and promoted alcohol-associated inflammatory and cancer-related pathways in colonic cecum mucosa. To elucidate possible mechanisms, we assessed the interactions of alcohol with WTF on colonic fecal microbiota that was in direct contact with the colonic cecum mucosa and found that colonic gene alterations coincided with altered colonic microbiota compositions. Our modeling indicated that altered colonic gene expression, in part, could be explained by the microbial changes imposed by WTF and alcohol.

Change of the food timing by WTF affected colonic circadian rhythm pathways in our study, consistent with the prior observations that time of eating controlled circadian pathways in the peripheral tissue including the intestine (8, 9, 21). Wrong-time feeding, when combined with alcohol, promoted dysregulation of a number of inflammatory and cancer-related pathways in the colon, associated with alcohol intake. In line with accumulating evidence suggesting the role of microbiota in CRC (50, 51) and its response effects in circadian disruption and alcohol intake (11, 21, 49), our model tested the interactions of alcohol and WTF on the changes in colonic microbiota. Although alcohol consumption alone affected α and β diversity of the gut microbiota, the effect was more pronounced in WTF mice. In the absence of WTF, alcohol consumption was not sufficient to induce a significant shift in the overall gut microbiota composition, in comparison to control mice. A similar interaction was seen in the colonic transcriptome, where WTF augmented alcohol's effect on promoting pathways related to CRC including those cancer-, metabolism-, and immune-related.

This study further adds to the accumulating literature on the aggravating effect of circadian disruption on the gut microbiota community structure and composition, from chronic alcohol consumption (20, 21). For the microbiota analysis of the current study, we used cecal content that was in close contact to the section used for transcriptome studies. The interaction of alcohol and circadian disruption induced by WTF was detected at multiple parameters of the colonic microbiota including α and β diversity and community structure. Comparison of the overall composition between alcohol consumption and control was significant only when RTF was present. Previous studies supported the interaction of other lifestyle habits with alcohol consumption on the alterations in the gut microbiota (52, 53).

Interestingly, WTF alone did not result in significant changes in the microbiota composition in the absence of alcohol. This is consistent with the overall hypothesis that circadian disruption alone may not be sufficient to cause deleterious effects in the intestine, but predisposes the host to pathologies when combined with another lifestyle stressor [i.e., alcohol, high fat diet, etc.; (11, 20, 54)]. From an ecological perspective, the impact of stressor on the microbial community depends on the initial status of the microbiota composition and its inherent stability and resilience (55). On the other hand, the type and frequency of a disturbance are important in determining the ecological response over time (56). Short-term (pulse) disturbance of a stable and resilient ecosystem frequently results in a transient alternative steady state, which is rapidly recovered following the termination of the disturbance (57). Chronic alcohol consumption could be regarded as a long-term (persistent) ecological disturbance, which resulted in the state transition of the colonic microbiota composition, following the colonic circadian disruption induced by WTF.

Synergism between alcohol consumption and WTF was also observed on intestinal gene expression alterations. Wrong-time feeding in alcohol-treated mice resulted in overexpression of Sal-like protein 3 [Sall3, a zinc finger–containing putative transcription factor implicated in innate lymphoid cell differentiation (58)] and α-defensin 24 (Defa24), whereas tight junction protein Cldn8 was down-regulated. Consequently, highly significant enrichment of immune-related and metabolism pathways was present.

We again observed that WTF alone mainly affected the expression of genes associated with circadian clock pathway. Approximately 30% of gene expression of intestinal epithelium undergoes diurnal variation (59). We hypothesized that WTF, although it does not cause a global gene dysregulation in the colon, through a circadian clock dysregulation may affect the diurnal variation of genes that are relevant in response to stressors such as alcohol. In the presence of alcohol, this resulted in exaggerated gene dysregulation. Future studies are needed to test this hypothesis.

Overall, we observed a stronger compositional shift in the colon microbiota, as well as gene dysregulation due to concomitant alcohol consumption and WTF. However, the causal direction of the changes between the alterations in the microbiota and transcriptome could not be directly inferred from our study. We modeled the degree of changes in colon gene expression that could be related to the changes by the microbiota induced by our treatments. Among the 13,782 genes, 3,999 (~third) had at least one paired microbiome significance. As a proof of concept to our approach, we found that Reg3a (regenerating family member 3α), which encodes an antimicrobial protein and mediates the effect of intestinal microbial content on the host immune response, demonstrated a relatively stable relationship with 10 microbiome taxa under our assumptions. In order to identify microbial taxa–gene relation, we employed the special random correspondence to obtain a more reliable and stringent result from our modeling. Our analyses suggested that changes in almost a third of our testable genes were significantly linked to the colonic microbial alterations associated with the treatments (alcohol and WTF). These findings suggest that altered colonic gene expression may in part be related to the microbial changes imposed by WTE and alcohol. While alcohol and circadian rhythm each can affect the intestinal gene expression (15, 60–63), the effect of gut microbiota on intestinal gene expression has been mostly studied in germ-free animal models or following the eradication of the bacteria by broad-spectrum antibiotic cocktail (64, 65).

In summary, in a colon-specific polyposis model, we observed that eating at rest affects circadian pathways in the colon and interacts with alcohol in changing colonic microbiota and transcriptome, resulting in up-regulation of CRC-related pathways. This is consistent with our recent studies that showed the promoting effect of circadian disruption on alcohol-induced polyposis could be microbiota-dependent (21). Further mechanistic studies could identify targetable mechanisms (i.e., microbiota) to mitigate the deleterious effects of lifestyle-associated risk factors on colon carcinogenesis (66). Studies are underway to extend these findings to human cohorts.

## Data Availability Statement

The datasets generated for this study can be found in the accession number of repository 16S rRNA microbiota sequence data: PRJNA523141, RNAseq sequence data: PRJNA588791.

## Ethics Statement

This animal study was reviewed and approved by Rush University Medical Center (IACUC # 14-008).

## Author Contributions

FB designed and supervised the study, and wrote the manuscript. PE, XL, YZ, and SM prepared the dataset, performed the statistical analysis, and analyzed the results. PE and SM helped in writing the manuscript. All authors have read and approved the final manuscripts.

## Conflict of Interest

The authors declare that the research was conducted in the absence of any commercial or financial relationships that could be construed as a potential conflict of interest.

## Supplementary Material

Figure S1 Polyp number count differences. TS4/APC mice were treated with Chow diet under two food timing conditions (right-time feeding/RTF during the dark vs. wrong-time feeding/WTF during the light) with or without 15% alcohol. In comparison to water treated animals, WTF with alcohol resulted in overall more neoplastic polyps (tubular adenomas) in the large and small intestine. ^*^*P* ≤ 0.05.

Figure S2 Total operational taxonomic unit reads per sample. **(A)** Total number of all OTUs (*n* = 47,568); **(B)** OTUs >5 reads in total (*n* = 1,564); **(C)** the number of OTUs >0.01% (*n* = 305); **(D)** the total number of OTUs >1% (*n* = 15).

Figure S3 Alcohol or wrong time feeding cecum mucosa gene expression levels. **(A)** Alcohol + RTF resulted in two-fold down-regulation of *Clps* (colipase), *Cpa2* (RNA polymerase II C-terminal domain phosphatase-like 2 in Arabidopsis), *Frs3* (Fibroblast growth factor receptor substrate 3), and up-regulation of *4930552P12Rik* (lincRNA); **(B)** WTF alone resulted in significant up-regulation of *Ciart* (Circadian-associated transcriptional repressor) and *Mterf1a* (Transcription termination factor 1a, mitochondrial). **(C)** WTF was sufficient to alter *Ciart* gene expression in comparison to alcohol + RTF; **(D)** WTF results in up-regulations of *Hoxa9* (Homeobox A9), *Six2* (SIX Homeobox 2), *Hoxb13* (Homeobox B13), *Hoxa6* (Homeobox A6), and down-regulation of *Apoc1* (Apolipoprotein C1), when compared to alcohol + WTF.

Figure S4 Pathway analysis of the most dysregulated pathways. Differentially expressed genes (DEGS) between treatment groups: **(A)** WTF vs. RTF; **(B)** alcohol + WTF vs. RTF; and **(C)** alcohol + WTF vs. alcohol + RTF.

Figure S5 Alpha diversity indices. Alpha diversity's observed OTUs and Inverse Simpson Index indicated no significant effect of alcohol consumption **(A,C)** or food timing alone **(B,D)** on the microbiota. *P*-values depicted within graph.

Table S1 Significant individual taxa at the phylum, family, and genus taxonomic levels.

Table S2 Among the 13,782 gene, 3,999 genes have at least one paired microbiome significance. 13,782-row (number of selected gene) and 34-column (number of selected microbiomes).

Table S3 Six taxa (red) were found to be strongly altered by the interaction of alcohol + WTF, while only three taxa (blue) were altered due to each of the factors alone.
