# Supplementary figures and images for: Abnormal Food Timing Promotes Alcohol-Associated Dysbiosis and Colon Carcinogenesis Pathways
Source: Front Oncol. 2020 Jul 17;10:1029. doi: 10.3389/fonc.2020.01029 (PMC7396506; doi:10.3389/fonc.2020.01029)

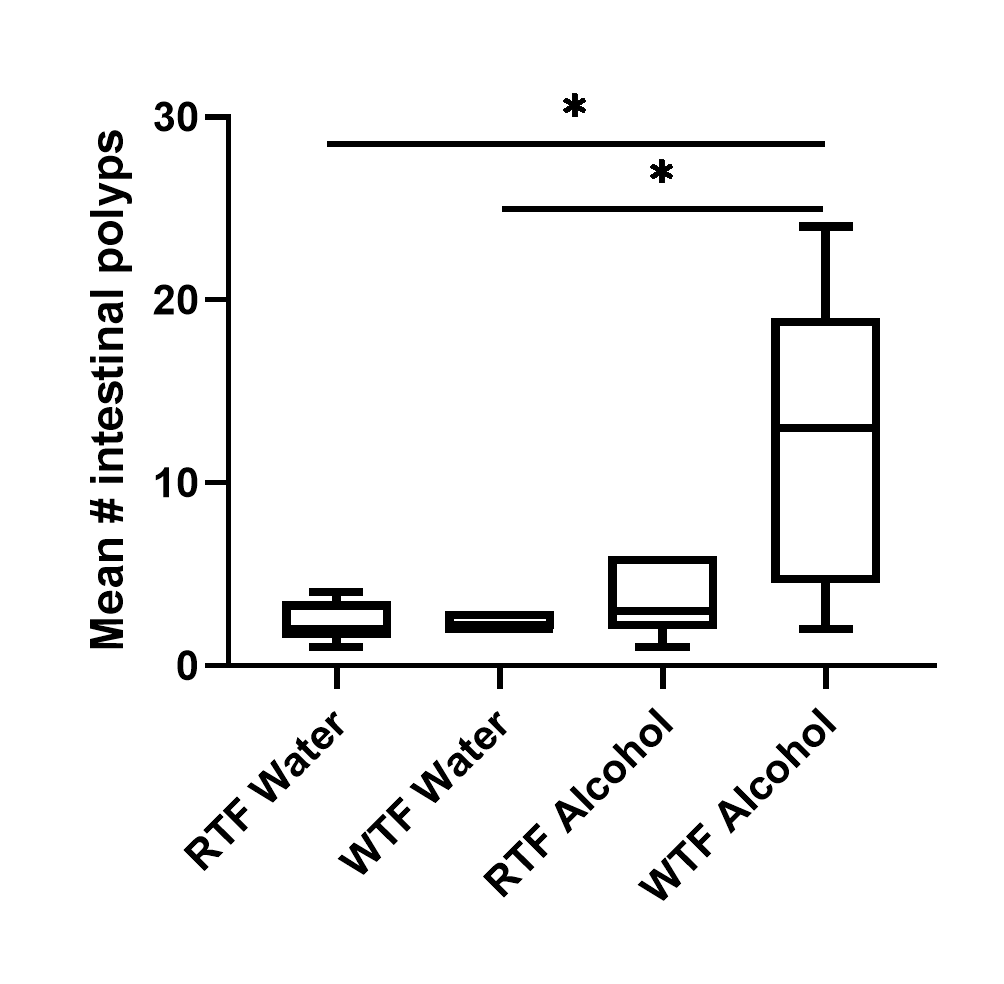

Supplement: Figure S1 — Polyp number count differences. TS4/APC mice were treated with Chow diet under two food timing conditions (right-time feeding/RTF during the dark vs. wrong-time feeding/WTF during the light) with or without 15% alcohol. In comparison to water treated animals, WTF with alcohol resulted in overall more neoplastic polyps (tubular adenomas) in the large and small intestine. *P ≤ 0.05. [file Image_1.TIF]

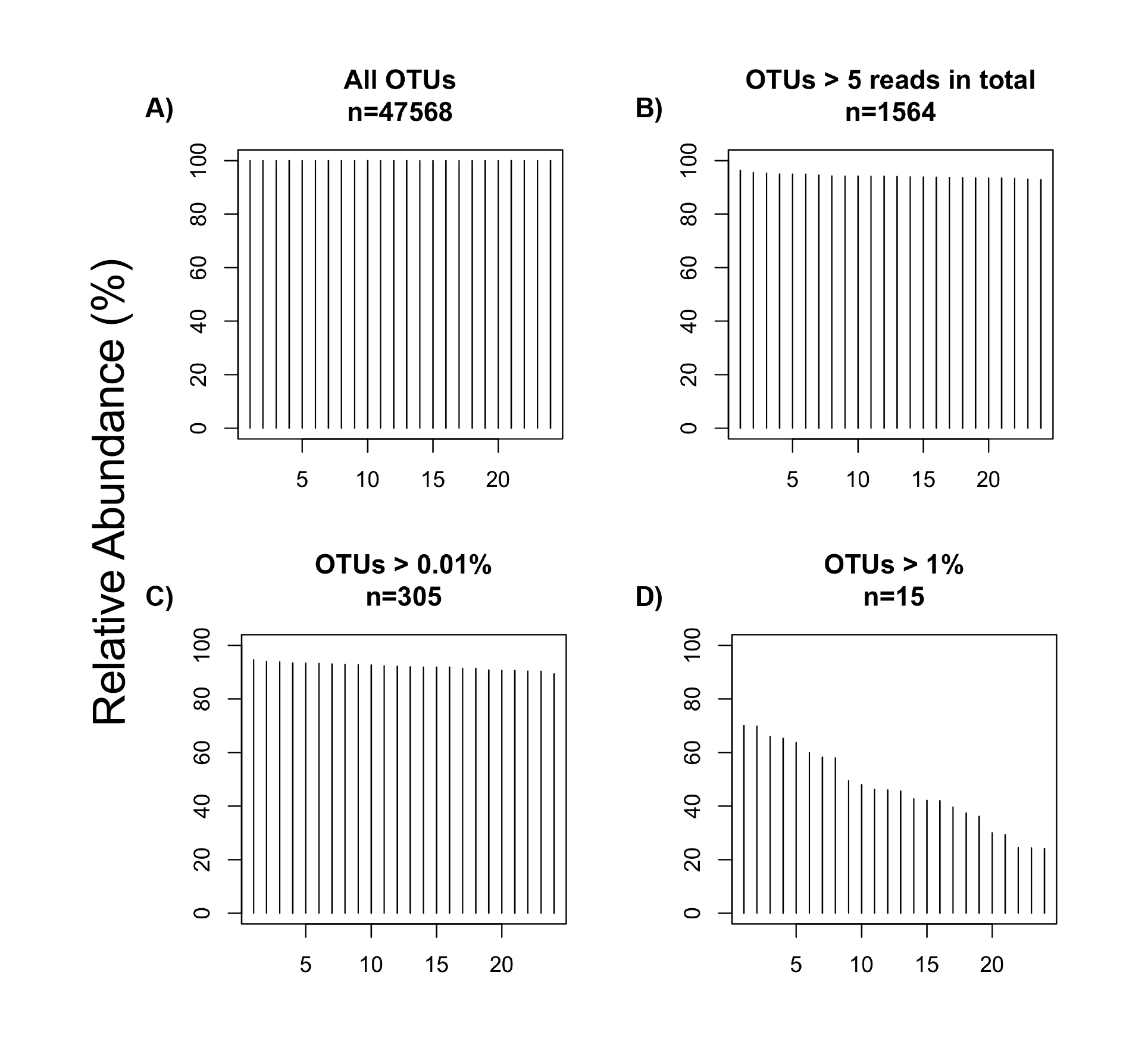

Supplement: Figure S2 — Total operational taxonomic unit reads per sample. (A) Total number of all OTUs (n = 47,568); (B) OTUs >5 reads in total (n = 1,564); (C) the number of OTUs >0.01% (n = 305); (D) the total number of OTUs >1% (n = 15). [file Image_2.TIF]

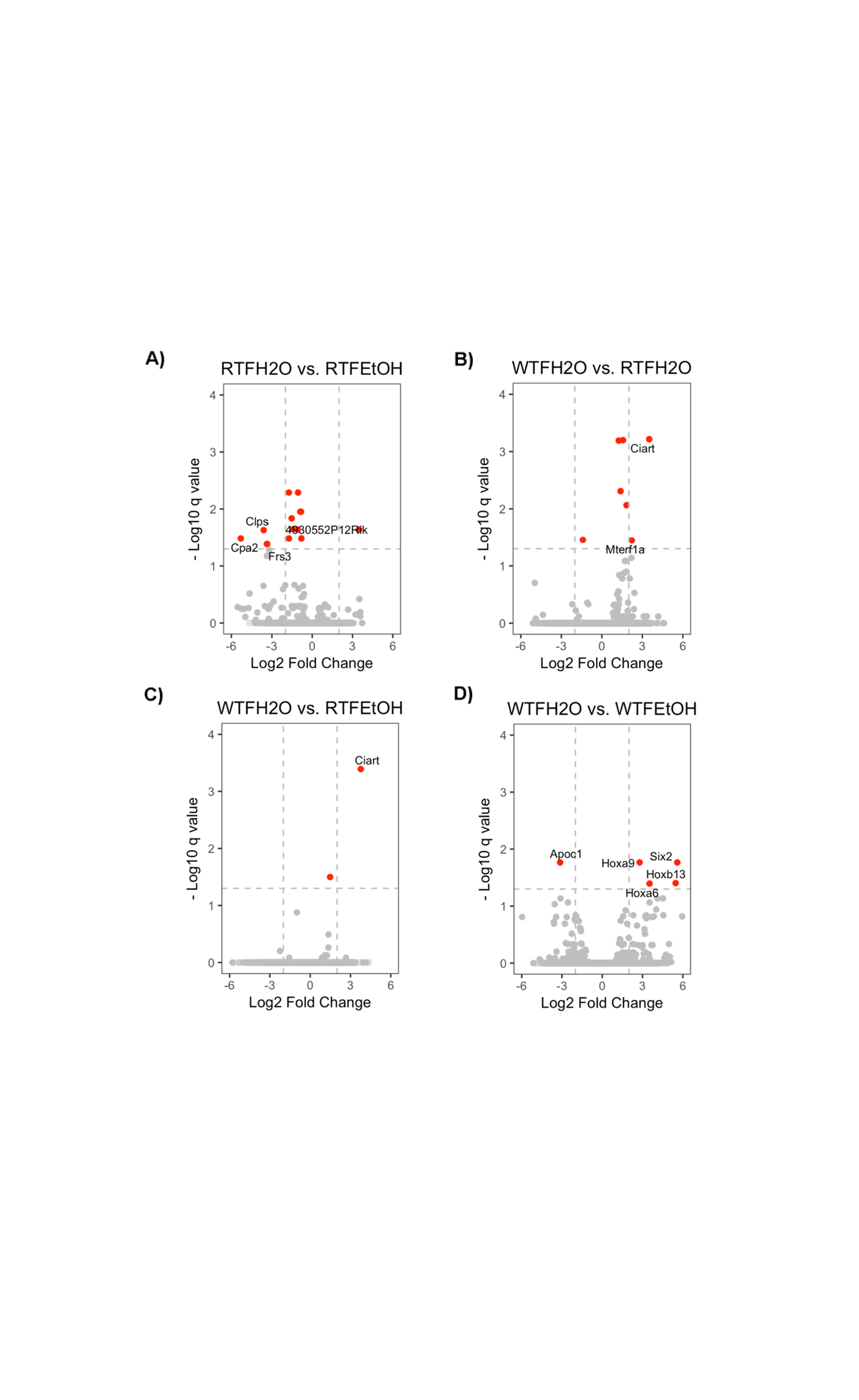

Supplement: Figure S3 — Alcohol or wrong time feeding cecum mucosa gene expression levels. (A) Alcohol + RTF resulted in two-fold down-regulation of Clps (colipase), Cpa2 (RNA polymerase II C-terminal domain phosphatase-like 2 in Arabidopsis), Frs3 (Fibroblast growth factor receptor substrate 3), and up-regulation of 4930552P12Rik (lincRNA); (B) WTF alone resulted in significant up-regulation of Ciart (Circadian-associated transcriptional repressor) and Mterf1a (Transcription termination factor 1a, mitochondrial). (C) WTF was sufficient to alter Ciart gene expression in comparison to alcohol + RTF; (D) WTF results in up-regulations of Hoxa9 (Homeobox A9), Six2 (SIX Homeobox 2), Hoxb13 (Homeobox B13), Hoxa6 (Homeobox A6), and down-regulation of Apoc1 (Apolipoprotein C1), when compared to alcohol + WTF. [file Image_3.TIF]

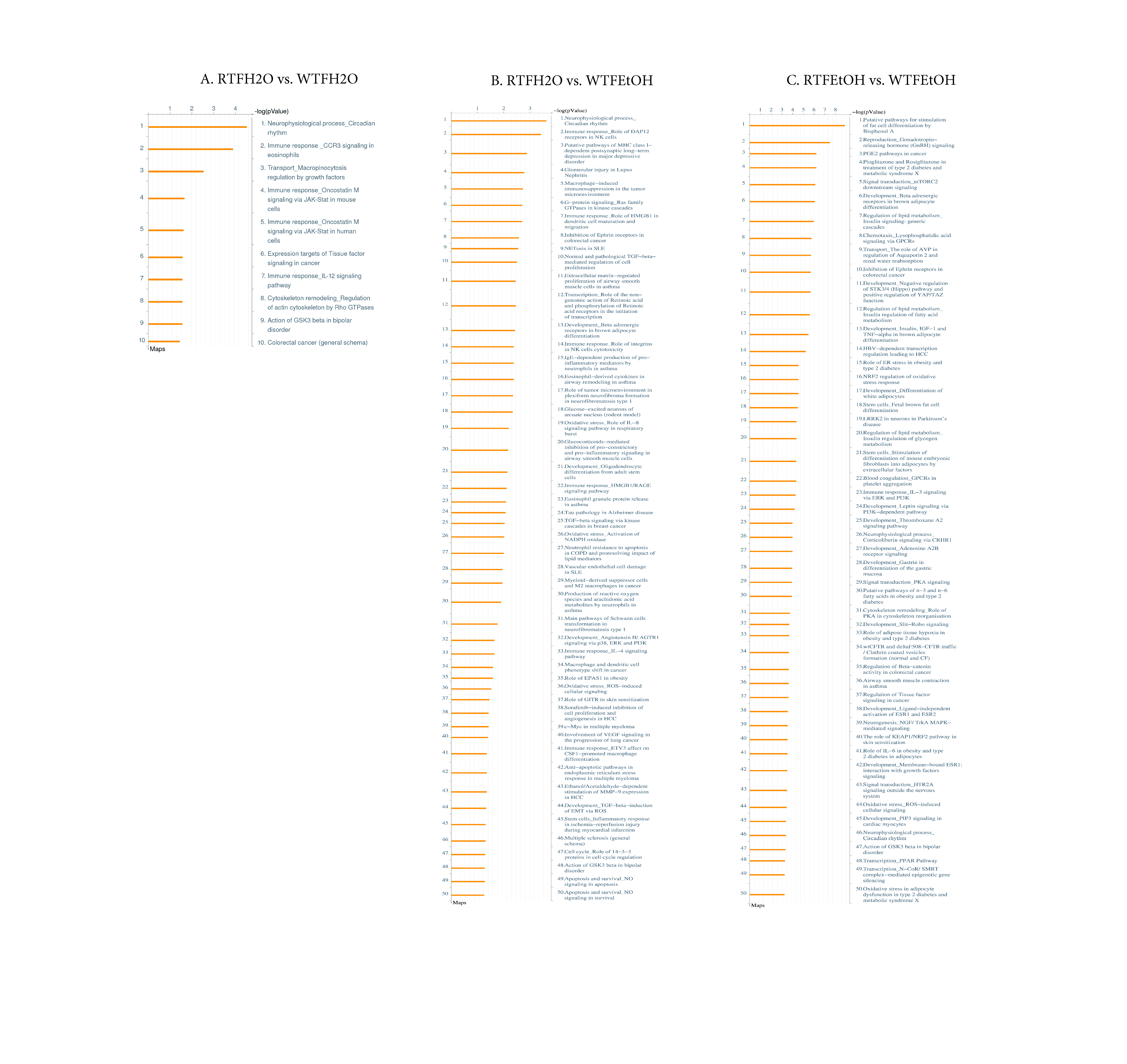

Supplement: Figure S4 — Pathway analysis of the most dysregulated pathways. Differentially expressed genes (DEGS) between treatment groups: (A) WTF vs. RTF; (B) alcohol + WTF vs. RTF; and (C) alcohol + WTF vs. alcohol + RTF. [file Image_4.TIFF]

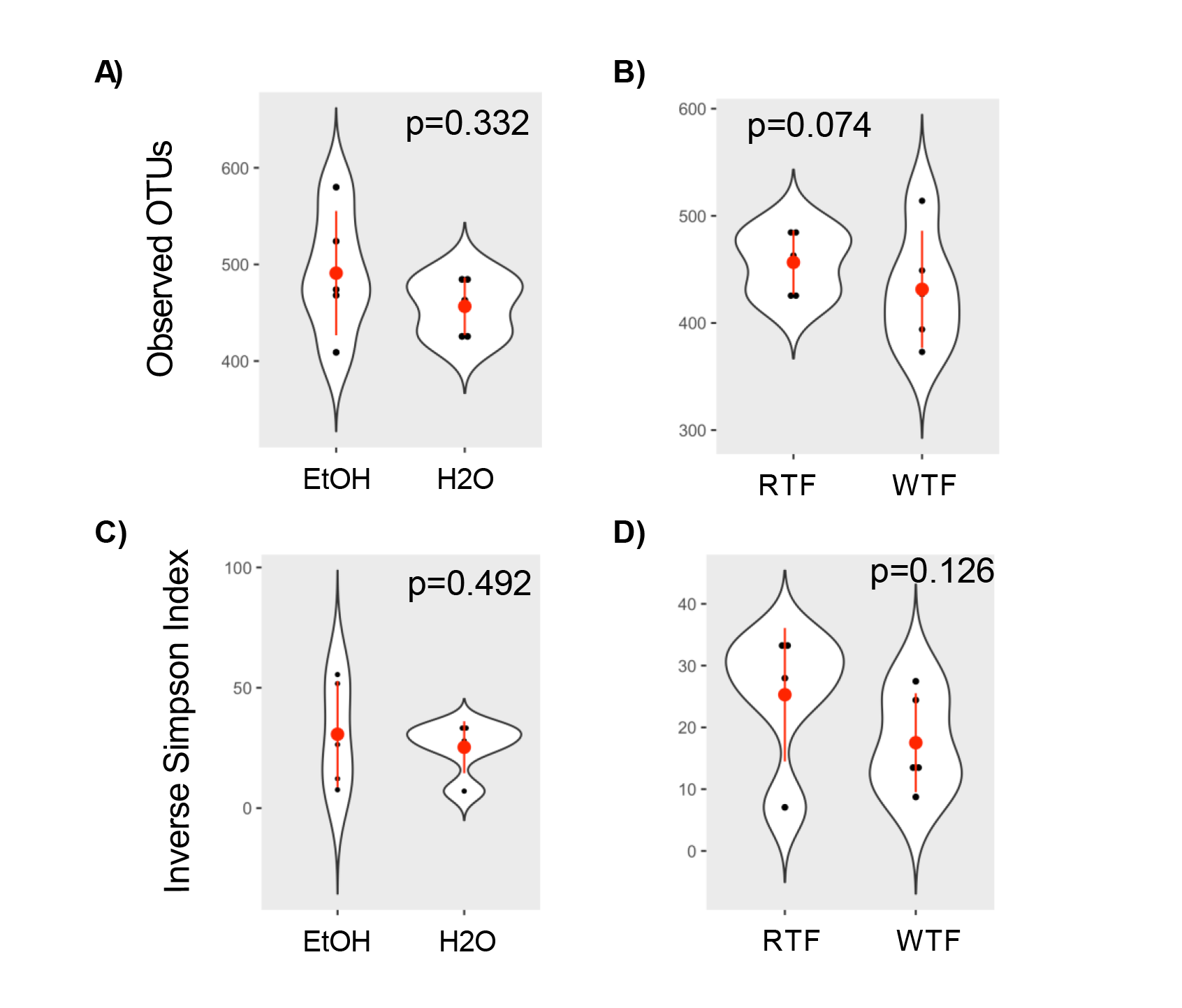

Supplement: Figure S5 — Alpha diversity indices. Alpha diversity's observed OTUs and Inverse Simpson Index indicated no significant effect of alcohol consumption (A,C) or food timing alone (B,D) on the microbiota. P-values depicted within graph. [file Image_5.TIF]
